# A Tale of Two Ends: Repurposing Metallic Compounds from Anti-Tumour Agents to Effective Antibacterial Activity

**DOI:** 10.3390/antibiotics9060321

**Published:** 2020-06-11

**Authors:** Daniela Alves Ferreira, Luísa M. D. R. S. Martins, Alexandra R. Fernandes, Marta Martins

**Affiliations:** 1 Department of Microbiology, Moyne Institute of Preventive Medicine, School of Genetics and Microbiology, Trinity College Dublin, the University of Dublin, College Green, Dublin 2, D02PN40, Ireland; alvesfed@tcd.ie; 2 Centro de Química Estrutural and Departamento de Engenharia Química, Instituto Superior Técnico, Universidade de Lisboa, 1049-001 Lisbon, Portugal; luisamargaridamartins@tecnico.ulisboa.pt; 3 UCIBIO, Departamento Ciências da Vida, Faculdade de Ciências e Tecnologia, Campus de Caparica, 2829-516 Caparica, Portugal

**Keywords:** antimicrobial resistance, drug repurposing, metallic compounds

## Abstract

The rise in antibiotic resistance coupled with the gap in the discovery of active molecules has driven the need for more effective antimicrobials while focusing the attention into the repurpose of already existing drugs. Here, we evaluated the potential antibacterial activity of one cobalt and two zinc metallic compounds previously reported as having anticancer properties. Compounds were tested against a range of Gram-positive and -negative bacteria. The determination of the minimum inhibitory and bactericidal concentrations (MIC/MBC) of the drugs were used to assess their potential antibacterial activity and their effect on bacterial growth. Motility assays were conducted by exposing the bacteria to sub-MIC of each of the compounds. The effect of sub-MIC of the compounds on the membrane permeability was measured by ethidium bromide (EtBr) accumulation assay. Cell viability assays were performed in human cells. Compound TS262 was the most active against the range of bacteria tested. No effect was observed on the motility or accumulation of EtBr for any of the bacteria tested. Cell viability assays demonstrated that the compounds showed a decrease in cell viability at the MIC. These results are promising, and further studies on these compounds can lead to the development of new effective antimicrobials.

## 1. Introduction

According to the World Health Organization (WHO), antimicrobial resistance (AMR) is one of the most complex threats in the 21st century, not only to human health but also to economics. The extensive misuse of antibiotics, among other factors, has contributed to the emergence of multidrug resistant bacteria, consequently leading to the development of hard-to-treat infections. Jim O’Neil and his team estimated that annual deaths attributable to antimicrobial resistant infections would reach approximately 10 million cases each year by 2050 unless action is taken [1]. Adding to this prediction, the European Centre for Disease Prevention and Control reported that 33,000 deaths occur each year in the European Union/European Economic Area (EU/EEA), mostly due to bacterial infections [2].

The discovery and development of novel antibiotics is stagnating and the urge for new approaches is increasing to meet the challenges posed by the rapid development of bacterial infections becoming resistant to common antimicrobial drugs. Several novel approaches benchmarked against the use of conventional antibiotic treatments, such as the use of combination of antibiotics [3], bacteriophage therapy [4], antibacterial antibodies [5], probiotics [6], nanomaterials [7], and antimicrobial peptides [8], among others, have been tested. Adding to this, the repurposing of existing drugs is becoming more attractive as a new strategy to fight AMR. This approach presents some advantages, namely, because the leverage of these drugs has already been approved for use in humans, and therefore this reduces the timelines required for the drugs to be effectively available for treatment. The most successful and classical case is sildenafil (brand names: Revatio, Viagra). This drug was originally designed to treat hypertension and then repurposed to treat erectile dysfunction and pulmonary arterial hypertension. In cancer therapy, this strategy has been advantageous in accessing previously approved non-cancer drugs to be directed for cancer treatment [9]. Therefore, the repurposing of anti-tumour compounds such as metallic compounds [10] could be of interest as a source of new antibacterials. Although the research of these compounds is mainly focused on antitumor purposes, to our knowledge, data available on the antimicrobial activity of these compounds is quite limited. An example of a very active antimicrobial compound described a long time ago and used for the treatment of syphilis is salvarsan, an organoarsenical compound [11].

In the present study, zinc and cobalt metallic compounds, with previously reported anti-tumour properties [12,13,14,15], were tested for their potential antimicrobial activity against a range of bacteria. These zinc and cobalt metallic compounds were selected from an initial screening of 17 new molecules synthesised and previously tested for their potential anti-tumour activity. From this initial screening for antimicrobial activity, three compounds (identified as TS262, TS265, and TS267) showed the best activity, i.e., lower minimum inhibitory concentration (MIC) and minimum bactericidal concentration (MBC) values against Gram-negative and -positive bacteria. On the basis of these results, we decided to investigate these three TS compounds further regarding their potential mode of action on bacteria. For that, we tested the effect of these compounds on the growth kinetics, motility, and permeability of the bacteria. There are several studies published regarding the effect that metallic compounds have on the bacterial membrane, by inducing damage to this structure and indirectly collapsing efflux systems [16,17,18]. Some bacterial efflux systems are dependent on the proton motive force and therefore any compound acting on this gradient will have an impact on the bacteria itself [19,20]. If a compound affects the proton motive force it will also impact bacterial motility since bacterial flagella are driven by a rotary motor that uses free energy stored in the electrochemical proton gradient across the cytoplasmic membrane to do mechanical work [21]. Therefore, the assessment of bacterial phenotypes such as increased permeability of the bacterial membrane and motility can be of importance when analysing the potential mode of action of these metallic compounds.

## 2. Results

### 2.1. Antimicrobial Activity of the Anti-Tumour Agents 

The antimicrobial activity and effect on bacterial growth of three metallic compounds with Zn and Co as metal centre were assessed against a range of Gram-positive and -negative bacteria. The activity of the three metallic compounds against bacteria is summarised in Table 1. These compounds were initially selected from a group of 17 new molecules previously tested for their potential anti-tumour activity (for more details please see Table S1).

From the three compounds tested, the Zn compound TS262 was the most active against all the seven strains tested (MIC range from 0.9 (0.5 µg/mL) to 14.4 µM–8 µg/mL). The compound that showed lower antibacterial activity was TS265 (MIC range from 1.6 (1 µg/mL) to 25.8 µM (16 µg/mL)). TS262 showed good activity against *Acinetobacter baumannii* ATCC19606 (MIC of 0.9 µM–0.5 µg/mL). The other two compounds, TS265 and TS267, also showed good activity against this bacterium, with MIC of 1.6 µM–1 µg/mL and 1.8 µM–1µg/mL, respectively. All compounds showed good activity against *A. baumannii* ATCC19606, with low concentrations not only inhibiting bacteria growth but also inducing its complete killing, indicating that they also have a good bactericidal activity. The three compounds were less active against *Klebsiella pneumoniae* and *Pseudomonas aeruginosa*, which is not totally unexpected, since Gram-negative bacteria are almost impermeable to new drugs because majority of these drugs are unable to penetrate the bacterial cell wall. Adding to this, they also possess other mechanisms of resistance and efflux systems that are involved in the extrusion of the drugs from the cell to the external environment. In general, the majority of the compounds showed bactericidal activity at the same level as the MIC, with the exceptions of TS262 tested against *A. baumannii* (MIC = 0.9 µM–0.5 µg/mL and MBC = 1.8 µM–1 µg/mL) and TS267 against *P. aeruginosa* (MIC = 14 µM–8 µg/mL and MBC = 328 µM–16 µg/mL).

**Figure 1:**
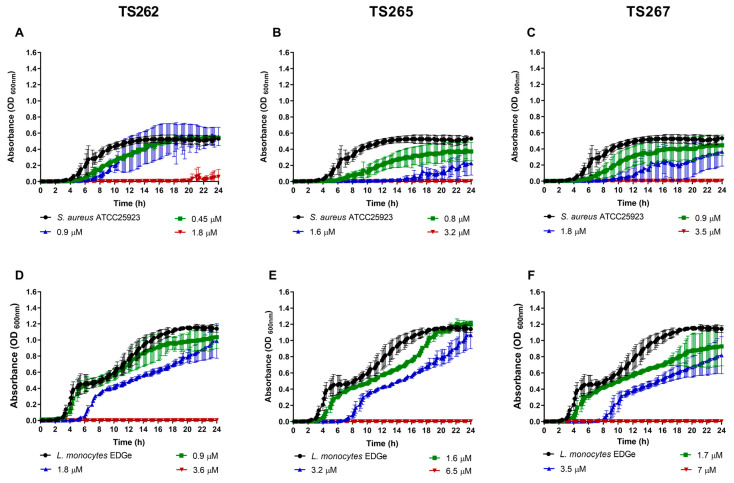
Growth kinetics of Gram-positive bacteria in the presence of the three metallic compounds. Effect of the metallic compounds on the growth of *S. aureus* ATCC25923 (**A**–**C**) and *Listeria monocytogenes* EDGe (**D**–**F**) in Mueller–Hinton (MH) broth. The results correspond to the average of three independent experiments ± standard deviation (SD).

**Figure 2:**
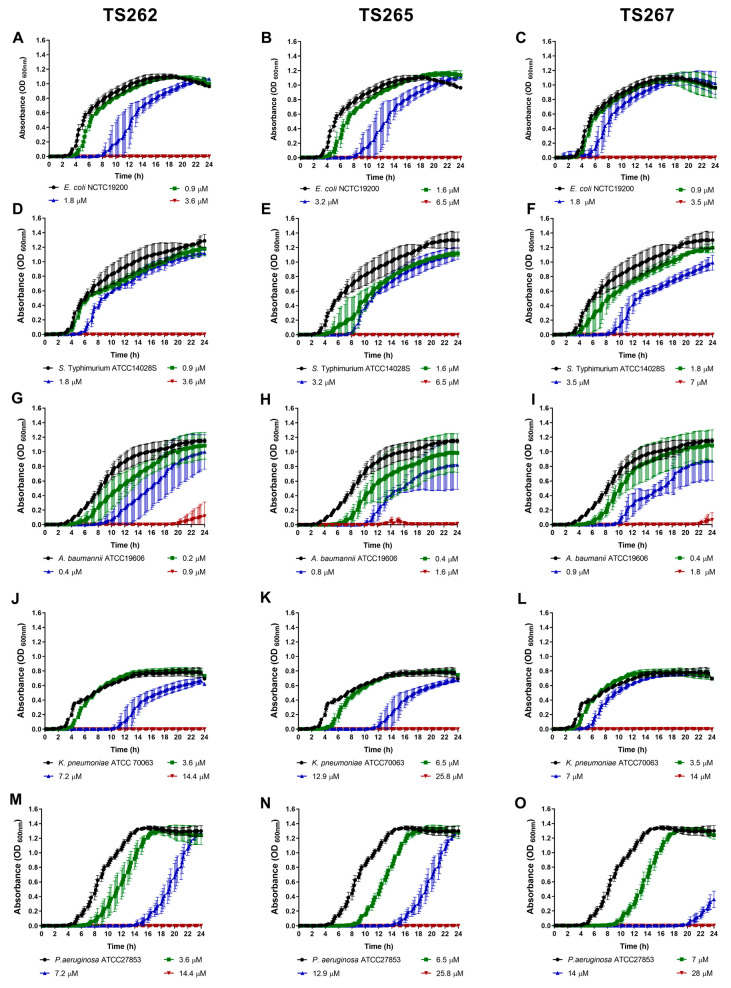
Growth kinetics of Gram-negative bacteria in the presence of the three metallic compounds. Effect of metallic compounds on the growth of *E. coli* (**A**–**C**), *S.* Typhimurium (**D**–**F**), *A. baumannii* (**G**–**I**), *K. pneumoniae* (**J**–**L**), and *P. aeruginosa* (**M**–**O**) in MH broth. The results presented correspond to the average of three independent experiments ± standard deviation (SD).

The growth of the bacterial strains in the presence of the MIC and sub-MICs of the compounds was monitored in rich media, namely, Mueller–Hinton (MH) broth (Figure 1 and Figure 2). Data obtained for Gram-positive bacteria (Figure 1A–F) indicated that growth was similar to the untreated (control) at the lowest concentration tested (corresponding to one-quarter MIC of each strain tested) of each compound, with no significant changes being observed, with the exception of *Staphylococcus aureus* (Figure 1A–C). A slight delay on the lag phase was observed when *S. aureus* was cultured in the presence of one-quarter MIC of the three compounds (Table 2). Concerning the Gram-negative bacteria (Figure 2A–O), a similar effect was observed, with the exception of *P. aeruginosa* (Figure 2M–O), where a slight extension of the lag phase was observed when the bacteria were grown in the presence of the three compounds. At one-half MIC of the three compounds, all the strains demonstrated an extension of the lag phase (Table 2). This effect was also observed on the Gram-negative bacteria (Figure 2A–O). This effect is not unexpected since it is known that bacteria require time to adapt to the presence of toxic compounds, in this case, the anti-tumour compounds. 

### 2.2. Effect on Bacterial Motility 

**Figure 3:**
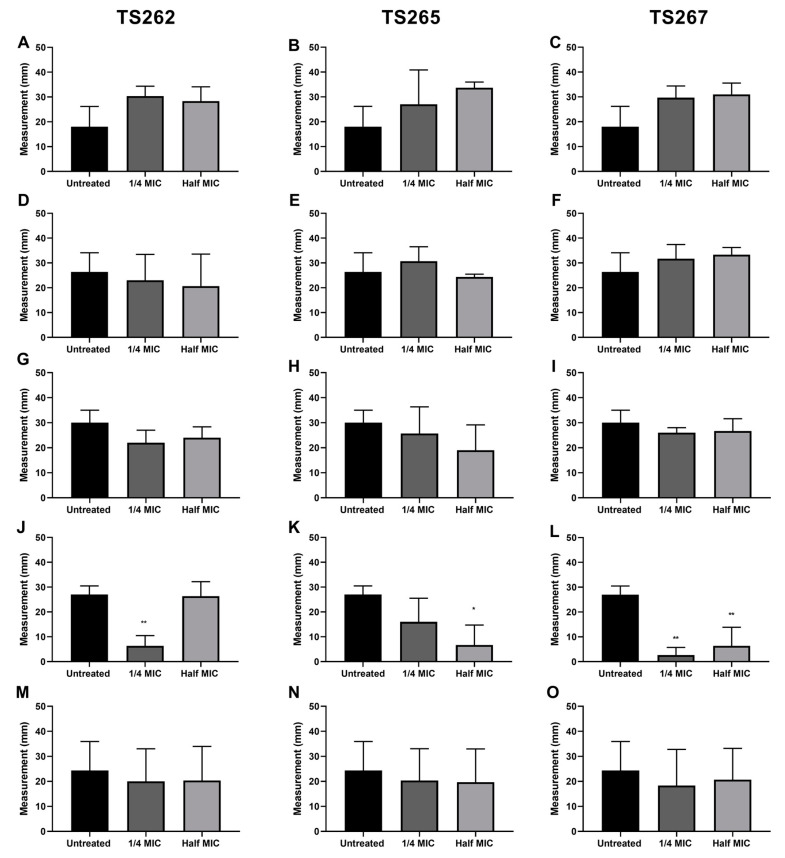
Effect of the anti-tumour compounds on bacterial swimming. The motility of *L. monocytogenes* (**A**–**C**), *E. coli* (**D**–**F**), *S.* Typhimurium (**G**–**I**), *A. baumannii* (**J**–**L**), and *P. aeruginosa* (**M**–**O**) was assessed in 0.4% MH agar. The results presented correspond to the average of three independent experiments ± standard deviation (SD). * *p* < 0.005 was considered significant and highly significant when ** *p* < 0.01 (one-way ANOVA test).

The swimming activity phenotype of each strain was assessed in the presence of TS262 (Figure 3A,D,G,J,M), TS265 (Figure 3B,E,H,K,N), and TS267 (Figure 3C,F,I,L,O). When the swimming activity of *A. baumannii* was tested in the presence of TS265 and TS267, a significant reduction on the swim activity of this strain was obtained, when compared to the untreated control. In the presence of any/each of the three compounds, *L. monocytogenes* showed a slightly higher swim activity (but not significant) when compared with the untreated bacteria. For the other strains, no effect was obtained when in the presence of the compounds in comparison with the untreated strains. *K. pneumoniae* and *S. aureus* strains were not included in this experiment due to lack of classical motility ability. 

### 2.3. Effect on Bacterial Membrane Permeability 

The seven strains were tested for their ability to accumulate ethidium bromide (EtBr) in the presence of sub-MIC of each TS compound. EtBr has been used to assess permeability of the bacterial membrane. It is a DNA-intercalating agent that enters and binds the DNA when the bacterial membrane integrity is compromised, and this results in an increase in the fluorescence emitted. Initially, the concentration of EtBr at which the influx of the dye equals its efflux (equilibrium) and does not affect bacterial viability was determined. The accumulation of EtBr started at concentrations above 0.315 µM for *L. monocytogenes, S. aureus*, and *K. pneumoniae;* 0.625 µM for *E. coli*, *P. aeruginosa*, and *S.* Typhimurium; and 1.25 µM for *A. baumannii* (Figure S1). Using these concentrations, determined previously, the potential of these three metallic compounds to permeabilise the membrane of Gram-positive and -negative bacteria at sub-inhibitory concentrations (one-half and one-quarter MIC) was assessed. Heat-inactivated bacteria were used as control for maximum fluorescence. When the bacteria were incubated with EtBr and the TS compounds (TS262, TS265, and TS267), we obtained no accumulation of EtBr (data not shown). These results indicate that the compounds do not affect the permeabilisation of the bacterial membrane either in Gram-positive or -negative bacteria. 

### 2.4. DNA–Metal Compound Interaction

**Figure 4:**
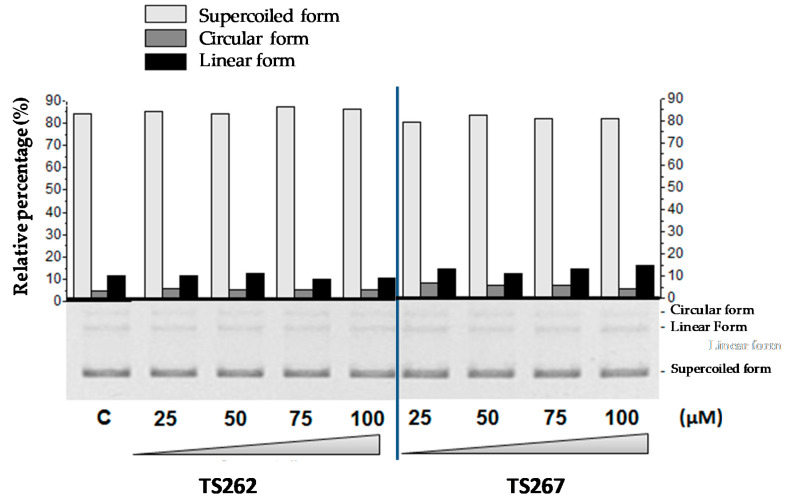
Electrophoretic evaluation of DNA double-strand cleavage by both Zn (II) phendione compounds (TS262 and TS267). Bottom: electrophoretic distribution of the three plasmid forms in an agarose gel (0.7%; *w/v*) as a result of exposure to 25–100 µM of the compounds. All reactions were conducted in 5 mM Tris-HCl and 50 mM NaCl (pH 7.02) for 24 h at 37 °C. Plasmid DNA pBluescript II SK(+) (pBSKII): (C) untreated. Top: densitometric quantification of plasmid forms using the image analysis software GelAnalyzer 2010a. Light grey bars represent the supercoiled form of plasmid DNA (pDNA), dark grey bars the circular form of pDNA, and black bars the linear form of pDNA. Results are expressed as mean ± SD of three independent experiments.

Previously, we demonstrated that TS265 was able to interact with DNA and induce plasmid DNA (pDNA) cleavage, as well as being able to produce double-strand breaks in a concentration-dependent manner [13]. In order to provide information concerning the capability of TS262 and TS267 to cleave pDNA, we incubated pDNA with increasing concentrations of each compound (separately) (Figure 4). As observed, neither compound was able to cleave pDNA or change the migration of supercoiled isoform, indicating that contrary to the Co(II) compound (TS265), these two Zn(II) compounds do not act in pDNA. 

### 2.5. Cell Viability

Due to the promising result shown by the TS262 compound concerning in vitro antimicrobial activity against Gram-positive and -negative bacteria, we additionally assessed its effect on the cell viability of human primary blood mononuclear cells (PBMC) and primary bronchial/tracheal epithelial cells (BTEC), and these results were compared to the inhibitory concentrations previously obtained in vitro. After 24 h of exposure, the viability of both cell lines was assessed using the Cell Titer 96^®^ AQueous One solution. Briefly, the conversion of a tetrazolium compound [3-(4,5-dimethylthiazol-2-yl)-5-(3-carboxymethoxyphenyl)-2-(4-sulfophenyl)-2H-tetrazolium, inner salt; MTS] into an aqueous formazan product is accomplished by dehydrogenase enzymes found in metabolically active cells [22].

**Figure 5:**
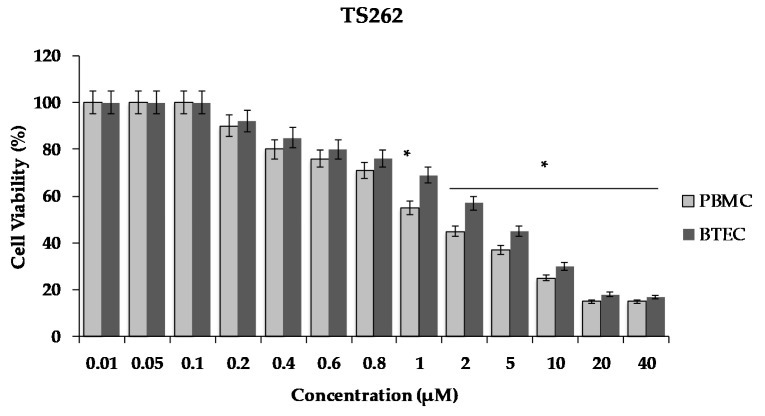
Effect of compound TS262 on the cellular viability of human primary blood mononuclear cells (PBMC) (black bar) and primary bronchial/tracheal epithelial cells (BTEC) (grey bar). Cells were exposed to the compound for 24 h at 37 °C, 5% CO_2_. The results presented correspond to the average of three independent experiments ± standard deviation (SD); percentage compared to controls from three independent biological replicates. Results were considered significant when * *p* < 0.01 (one-way ANOVA test).

As shown in Figure 5, there was a decrease of cell viability in a concentration dependent manner. The exposure of the cells to the Zn compound (TS262) at 2 µM caused a decrease in viability by 55%, only showing 45% of viable PBMC. When we analysed the effect in BTEC only for concentrations higher than 5 µM, a reduction of viability of 50% was observed (Figure 5). Indeed, at 5 µM, a decreased of viability was observed by 55% with 45% of viable BTEC. 

## 3. Discussion

The escalating numbers of antibiotic-resistant bacteria worldwide raises the urgency for novel classes of antimicrobial compounds. Treatment options that rely on existing antibiotics are becoming less effective, and therefore re-purposing of existing non-antibiotic compounds, such as metallic anti-tumour compounds, would be a valuable approach. Here, we demonstrated that zinc and cobalt compounds have in vitro antimicrobial activity against Gram-positive and -negative bacteria. These Zn and Co anti-tumour compounds were tested for their potential effect on the growth kinetics, motility, and permeability of the bacteria. In vitro, compound TS262 was the most active against all the seven strains tested with an MIC range from 0.9 to 14.4 µM. This compound showed good activity against *A. baumannii* ATCC19606, with an MIC of 0.9 µM. The other two compounds, TS265 and TS267, also showed good activity against this bacterium, with MIC of 1.6 µM and 1.8 µM, respectively. These results are very encouraging since *A. baumannii* is one of the main pathogens related to hospital-acquired infections and is considered a critical pathogen (in the case of resistance to carbapenems) in the WHO priority pathogens list for research and development of new antibiotics. When the swimming activity of *A. baumannii* was tested in the presence of TS265 and TS267, we obtained a significant reduction when compared to the untreated control. When these compounds were tested for their potential effect on the permeabilisation of the bacteria, we obtained no effect. Therefore, these Zn and Co anti-tumour compounds did not seem to damage the cell wall of Gram-positive or -negative bacteria, which is in opposition to other published studies that tested different compounds with effects on the bacterial membrane [11,16,23,24].

We have also shown that contrary to the Co (II) compound TS265, which was able to cleave DNA in a redox-dependent manner [13], Zn (II) compounds were not able to cleave pDNA. On the basis of these results, we can only speculate that the potential cellular targets of these compounds might be related with protein dysfunction and/or impaired enzyme activity due to oxidative protein damage or exchange of a structural or catalytic metal. Since Co (II) compound TS265 was able to cleave DNA in a redox-dependent manner, we speculate that the production of reactive oxygen species and antioxidant depletion demonstrated in various other metal toxicity studies, particularly in the case of compounds complexed with iron and copper, could be a possibility. We can speculate that due to the structure of these compounds, they could also interfere with nutrient uptake and assimilation by the bacteria, which can directly affect gene expression and signalling mechanisms of quorum sensing on the bacterial population [16,18,25]. 

One important aspect to be considered in the design of a new antibacterial is the possibility of the bacteria becoming resistant to these compounds through well-known mechanisms such as active efflux. To address this, we previously conducted efflux studies on *E. coli* in the presence and absence of these compounds at varying concentrations. These compounds showed no direct effect in the efflux of the bacteria (data not shown) and therefore we do not anticipate these compounds to be extruded from the bacterial cell, therefore giving rise to resistance. However, future studies will address this question in more detail by using bacterial strains that have well-characterised mechanism of resistance to metals, such as Hg and Cu, among others [26,27,28,29,30,31].

Considering the more interesting results of compound TS262, particularly with *A. baumannii* ATCC19606, we tested its effect on the viability of PBMC and BTEC normal primary cells. These compounds seem to have a negative effect on the viability of these cells at very low concentrations. However, it is important to stress that the concentrations needed to achieve a reduction of viability over 50% were 2 µM and 5 µM for PBMC and BTEC, respectively. Interestingly these results agree with previous data obtained by our group on normal epithelial cells [13]. Indeed, the half-maximal inhibitory concentration (IC_50_) obtained for this normal epithelial cell line (5.14 ± 0.01) µM is more than 6.9 times higher than that determined for a tumorigenic breast cell line MCF7 (0.73 µM) [13]. Considering the MIC of 0.9 µM, a therapeutic window might exist (of 2.5× for PBMC, 6.25× for BTEC, and 6.9× for epithelial cells). Nevertheless, for human administration, the vectorisation of these compounds using for instance gold nanoparticles [14] might be an interesting approach that we can consider for future work. 

Due to the promising activity that these compounds reported in vitro, they can be an alternative to the available arsenal of antibacterials. Additionally, these compounds can constitute good parental molecules to re-design derivatives of these molecules, presenting different metal centres. These approaches contribute to the highlighting of the fact that the repurposing of drugs can constitute a source for new antimicrobial compounds to fight infections caused by antibiotic-resistant bacteria.

## 4. Materials and Methods 

### 4.1. Compounds 

Three metallic compounds coupled with Zn and Co were used in this study. The compounds were identified as follows: [Zn(phendione)_2_] Cl_2_ (phendione = 1,10-phenanthroline-5,6-dione)—TS262; Co(II) coordination compound CoCl(H_2_O)(phendione)_2_][BF_4_] (phendione = 1,10-phenanthroline-5,6-dione)—TS265; and [ZnCl(κO-PTA = O)(phendione)]]BF_4_] (phendione = 1,10-phenanthroline-5,6-dione)—TS267 [12]. The main characteristics of the compounds are described in more detail in Table 3. These compounds were previously synthesised by Silva et al. [12], and their activity was screened against three human tumour cell lines.

### 4.2. Bacterial Strains

Listeria monocytogenes EGDe, Staphylococcus aureus ATCC25923, Acinetobacter baumannii ATCC19606, Escherichia coli NCTC19200, Klebsiella pneumoniae ATCC70063, Salmonella Typhimurium ATCC14028S, and Pseudomonas aeruginosa ATCC27853 were used in this study. The strains were grown in Mueller–Hinton (MH) broth at 37 °C with shaking.

### 4.3. Antibacterial Activity

The minimum inhibitory concentration (MIC) was determined by using the broth microdilution method in a 96-well plate according to the Clinical and Laboratory Standards Institute (CLSI) guidelines [32]. Briefly, overnight cultures were diluted in sterilised PBS to ≈10^5^ colony forming units (CFU)/mL. Aliquots of 10 µL were then transferred to separate wells in a 96-well plate that contained 100 µL of each compound at varying concentrations in Mueller–Hinton (MH) broth. Metallic compounds were tested at concentrations ranging from 0.2 µM to 103.6 µM (TS262), 0.2 µM to 103.3 µM (TS265), and 0.2 µM to 112 µM (TS267). After incubation at 37 °C for 18 h, we determined the MIC as the lowest concentration of compound where no visible growth of the bacteria was obtained, i.e., the lowest concentration of compound that was able to inhibit growth of the bacterial culture when the wells were read by eyeball. The determination of the minimum bactericidal concentration (MBC) was performed by replica transfer of the MIC plate into a new 96-well plate with compound-free media. Plates were incubated at 37 °C and the MBC results were recorded after 18 h. The assays were performed with three biological replicates. 

### 4.4. Bacterial Growth in the Presence of the Anti-Tumour Compounds

A 96-well plate was prepared with different concentrations of the three metallic compounds in Mueller–Hinton (MH) broth. Cultures of the bacteria that were diluted overnight (≈10^5^ colony forming units (CFU)/mL) were added to each well, except to control wells (sterility) where only media were added. The microplate was then incubated in a microplate reader (Synergy HT multi-mode; Bio-Tek, Winooski, VT, USA), and the bacterial absorbance (optical density; OD_600 nm_) was measured over a period of 24 h at 37 °C with shaking. The assays were performed with three biological replicates.

### 4.5. Motility Assays

To assess bacterial motility, we performed swim and swarm assays on all isolates in the absence/presence of the compounds, as previously described [33]. Briefly, for the swim assays, culture plates were previously prepared (Luria-Bertani (LB)) broth with 0.4% (*w/v*) agar; Sigma, Arklow, Ireland) and stab-inoculated with bacterial cultures grown overnight in LB broth. These plates were then incubated for 8 h at 37 °C. The region of visible colony spread on the agar was measured (in millimetres) with a ruler. Swarm motility assays were also performed with bacterial cultures grown overnight in LB broth. One microliter of each bacterial suspension was spotted directly onto a motility plate (LB broth with 0.6% (*w*/*v*) agar with 0.5% glucose; Sigma, Arklow, Ireland) and incubated at 37 °C overnight for 24 h. The region of visible colony spread on the agar was then recorded.

### 4.6. Membrane Permeability

Accumulation of ethidium bromide (EtBr) was measured as an indication of the ability of the compounds to permeabilise the bacterial membrane. To determine the EtBr concentration that did not exceed the ability of the efflux systems to extrude EtBr, we performed initial accumulation assays in the presence of increasing concentrations of EtBr, as previously described [34]. The bacterial strains were grown in MH broth until mid-log phase. Bacterial cells were washed with phosphate-buffered saline (PBS) solution and the OD was adjusted to 0.3 at 600 nm. Aliquots of this suspension with EtBr were transferred into a 96-well plate black flat bottom (Costar, Sigma-Aldrich, St. Louis, MO, USA). Heat-inactivated (30 min at 90 °C) bacteria were used as a control for maximum fluorescence. Fluorescence was recorded using a Synergy HT multimode microplate reader for 30 min using excitation and emission filters of 515 and 600 nm, respectively. The compounds were then added at sub-MIC. The technical duplicates were averaged and plotted, and were compared on the basis of their average fluorescence units. The assays were performed with three biological replicates.

### 4.7. Electrophorectic Analysis of DNA-Metal Compound Interaction

The interactions between DNA binding assays were conducted to determine if compound TS262 was able to cleave plasmid DNA. Plasmid DNA (pDNA) was obtained from *E. coli* transformed cells, and grown overnight (o.n.) in LB liquid medium (Applichem, Darmstadt, Germany) with 100 μg mL^−1^ ampicillin (Bioline, London, UK) at 37 °C with stirring. Plasmid extractions were performed using the Invisorb Spin Plasmid Mini Two Kit (Invitek, Berlin, Germany) and DNA were quantified by spectrophotometry with NanoDrop 2000 (Thermo Scientific, Waltham, MA, USA). The interactions between TS262 and pBluescript II SK(+) (pBSKII) DNA (Agilent Technologies, Santa Clara, CA, USA) were determined as previously described [13]. For the concentration-dependent studies, we incubated pBSK II (200 ng in a final volume of 20 µL) in the presence (25–100 µM) or absence of the tested compound for 24 h at 37 °C in reaction buffer (5 mM Tris-HCl, 50 mM NaCl (pH 7.02)).

### 4.8. Mammalian Cell Culture and Cell Viability

Primary bronchial/tracheal epithelial cells (BTEC) and primary peripheral blood mononuclear cells (PBMC) were purchased from the American Type Culture Collection (ATCC) (www.atcc.org) and cultured according to the manufacturer’s specifications. BTEC were maintained in Airway Epithelial Cell Basal medium (ATCC) supplemented with bronchial epithelial cell growth kit (ATCC), 33 μmol/L Phenol Red (Sigma), and a mixture of 100 U/mL penicillin and 100 μg/mL streptomycin (ThermoFisher Scientific, Waltham, MA, USA) at 37 °C and 5% CO_2_ (as described in [35]). PBMC have a limited lifespan in culture and should only be thawed immediately prior to their use and maintained in Roswell Park Memorial Institute (RPMI) 1640 (Sigma) supplemented with 10% foetal bovine serum (FBS; Sigma), 2 mM glutamine (Sigma), 1% nonessential amino acids (Sigma), 1% sodium pyruvate (Sigma), and 50 U/mL penicillin and streptomycin (ThermoFisher Scientific). One day before treatment, cells were seeded at a density of 2 × 10^4^ cells per well on a 96-well plate containing the respective media at 37 °C, 5% (*v*/*v*) of CO_2_, and an atmosphere of 99% (*v*/*v*) humidity for 24 h. Cell viability was assessed after exposure to concentrations between 0 and 40 µM of TS262. MTS assay (CellTiter 96 AQueous Non-Radioactive Cell Proliferation Assay, Promega, Madison, WI, USA) was performed 24 h after initial stimulus (at 37 °C) using 3-(4,5-dimethylthiazol-2-yl)-5-(3-carboxymethoxyphenyl)-2-(4-sulfophenyl)-2H-tetrazolium and inner salt (MTS) as previously described [36]. Briefly, MTS was added to each well and cells were incubated during 45 min at 37 °C. Following incubation, the absorbance of the soluble formazan product (brown) were measured in a microplate reader (TECAN, GE) at 490 nm. The formazan product is directly proportional to the number of living cells. The assays were performed with three biological replicates.

### 4.9. Statistical Analysis

Statistical analyses were performed using Prism Graphpad software version 8.0.2. *, **, ***, and **** represent *p*-values of <0.05, <0.01, <0.001, and <0.0001, respectively. *p* < 0.05 was considered statistically significant and highly significant when ** *p* < 0.01, *** *p* < 0.001, and **** *p* < 0.0001. One-way ANOVA was used to compare the mean values of at least three independent samples, where there was one independent variable in the experimental procedure, allowing for the determination of any statistically significant difference between the sample mean.

## Figures and Tables

**Table 1:** Antibacterial activity of metallic compounds against a range of Gram-positive and -negative bacteria.

Bacteria	TS262		TS265		TS267	
	MIC	MBC	MIC	MBC	MIC	MBC
	µM					
*Staphylococcus aureus* ATCC25923	1.8	1.8	3.2	3.2	3.5	3.5
*Listeria monocytogenes* EGDe	3.6	3.6	6.5	6.5	7.0	7.0
*Escherichia coli* NCTC12900	3.6	3.6	6.5	6.5	3.5	3.5
*Salmonella* Typhimurium ATCC14028S	3.6	3.6	6.5	6.5	7.0	7.0
*Acinetobacter baumannii* ATCC19606	0.9	1.8	1.6	1.6	1.8	1.8
*Klebsiella pneumoniae* ATCC70063	14.4	14.4	25.8	25.8	14	14
*Pseudomonas aeruginosa* ATCC27853	14.4	14.4	25.8	25.8	14	28

Legend: MIC, minimum inhibitory concentration; MBC, minimum bactericidal concentration; µM-micromolar. [Zn(phendione)_2_] Cl_2_ (phendione = 1,10-phenanthroline-5, 6-dione)—TS262; Co(II) coordination compound CoCl(H_2_O)(phendione)_2_][BF_4_] (phendione = 1, 10-phenanthroline-5, 6-dione)—TS265; and [ZnCl(κO-PTA = O)(phendione)]]BF_4_] (phendione = 1, 10-phenanthroline-5, 6-dione)—TS267.

**Table 2:** Summary of the effect of the three metallic compounds on the bacterial lag phase.

Bacterial Strains	Lag Phases (in hours)						
	Control	TS 262		TS 265		TS 267	
		¼MIC	½MIC	¼MIC	½MIC	¼MIC	½MIC
*S. aureus* ATCC25923	4.75	6	7.5	7.5	16.5	6.5	11.5
*L. monocytogenes* EGDe	3.25	3.2	6	4	7.3	3.75	8.25
*E . coli* NCTC12900	3	4.5	8.25	4.5	8.5	4	5.25
*S.* Typhimurium ATCC14028S	3.75	4	6	5	8	4	9
*A. baumannii* ATCC19606	3.75	5.25	9	7.25	10.5	6	10.25
*K. pneumoniae* ATCC70063	3	4.25	11.25	5	12	4	5.5
*P. aeruginosa* ATCC27853	5	8	15.25	9	15	-	10

**Table 3:** Chemical formula, molecular weight, and solvent of the three metallic compounds and their ligands.

Compound	Chemical Formula	Molecular Weight (g mol^−1^)	Solvent
**TS262**	C_24_H_12_Cl_2_N_4_O_4_Zn	556.67	H_2_O
**TS265**	C_24_H_15_BClCoF_4_N_4_O_5_	620.59	H_2_O
**TS267**	C_18_H_18_BClF_4_N_5_O_3_PZn	570.99	H_2_O
**DION (phendione)**	C_12_H_6_N_2_O_2_	210.19	H_2_O
**PTA (1,3,5-Triaza-7-phosphaadamantane)**	C_6_H_12_N_3_P	157.15	H_2_O

## Supplementary Materials

The following are available online https://www.mdpi.com/2079-6382/9/6/321, Figure S1: Determination of the equilibrium concentration of ethidium bromide (EtBr) in *S. aureus* (A), *L. monocytogenes* (B), *E. coli* (C), *S.* Typhimurium (D), *A. baumannii* (E), *K. pneumoniae* (F) and *P. aeruginosa* (G). Table S1: Antibacterial activity of metallic compounds against Gram -positive and -negative bacteria.
