# Supplementary material for: A Tale of Two Ends: Repurposing Metallic Compounds from Anti-Tumour Agents to Effective Antibacterial Activity
Source: Antibiotics (Basel). 2020 Jun 11;9(6):321. doi: 10.3390/antibiotics9060321 (PMC7344542; doi:10.3390/antibiotics9060321)
Supplement: Supplementary file 1 [file antibiotics-09-00321-s001.pdf]

A tale of two ends: repurposing metallic compounds from anti-tumor agents to effective antibacterial

Daniela Alves Ferreira[1], Luísa M.D.R.S. Martins[2], Alexandra R. Fernandes[3,*] and Marta Martins[1,*]

[1]Department of Microbiology, Moyne Institute of Preventive Medicine, School of Genetics and Microbiology, Trinity College Dublin, the University of Dublin, College Green, Dublin 2, D02PN40, Ireland.

[2] Centro de Química Estrutural and Departamento de Engenharia Química, Instituto Superior Técnico, Universidade de Lisboa, 1049-001 Lisbon, Portugal.

[3]UCIBIO, Departamento Ciências da Vida, Faculdade de Ciências e Tecnologia, Campus de Caparica, 2829-516 Caparica, Portugal.

* Correspondence: ma.fernandes@fct.unl.pt (A.R.F.); mmartins@tcd.ie (M.M.); Tel.: +351-212948530 (ext. 11107) (A.R.F.); +353-1-896-1194 (M.M.)

Supplementary Data

Table S1. Antibacterial activity of metallic compounds against Gram -positive and -negative bacteria.

Compound ID	Solvent	Metal	*S. aureus* (mg/L)		*E. coli* (mg/L)	
			MIC	MBC	MIC	MBC
**I.6**[a]	DMSO	Cu	32	64	>128	>128
**II.4**[a]	DMSO	Cu	>128	>128	>128	>128
**ComeOH**[a]	H_2O	Cu	>128	>128	>128	128
**TS119**[a]	H2O	Cu	>128	>128	>128	>128
**TS217**[b]	H_2O	Co(II)	>128	>128	>128	>128
**TS232**[c]	H_2O	Co	>128	>128	>128	>128
**TS236**[d]	DMSO	Co	>128	>128	>128	128
**TS253**[c]	H_2O	Co(II)	>128	>128	>128	>128
**TS254.1**[e]	H_2O	V	8	8	8	8
**TS262.1**[b]	H_2O	Zn(II)	**1**	**1**	**2**	**2**
**TS265.1**[b]	H_2O	Co(II)	**2**	**2**	**4**	**4**
**TS267.1**[b]	H_2O	Zn(II)	**2**	**2**	**2**	**2**
**TS293**[d]	DMSO	Co	>128	>128	>128	>128
**RTS218.1**[e]	H_2O	V	>128	>128	>128	>128
**JL653 F2**[f]	DMSO	Pt(II)	>128	>128	>128	>128
**ZM2**[a]	DMSO	Cu	32	32	64	64
**ZM5**[g]	DMSO	Cu	16	32	64	64

Legend: MIC, Minimum inhibitory concentration; MBC, Minimum bactericidal concentration. [Zn(phendione)2] Cl2 (phendione = 1,10-phenanthroline-5,6-dione) - TS262; Co(II) coordination compound CoCl(H2O)(phendione)2][BF4] (phendione = 1,10-phenanthroline-5,6-dione) - TS265 and [ZnCl(κO-PTA=O)(phendione)]]BF4] (phendione = 1,10-phenanthroline-5,6-dione) - TS267. Bacterial strains used were Methicillin Resistant *S. aureus* ATCC43300 and *E. coli* ATCC25922. The structures, details of the synthesis and characterization of all of the compounds were previously published (a-g) (please see references below).

(a) Ma, Z.; Zhang, B.; Guedes da Silva, M.F.C.; Silva, J.; Mendo, A.S.; Baptista, P.V.; Fernandes, A.R.; Pombeiro, A.J.L. 2016. Synthesis, characterization, thermal properties and antiproliferative potential of copper(ii) 4′-phenyl-terpyridine compounds. *Dalton Trans.* **2016**, 5339-5355.

(b) Silva, T.F.S.; Smoleński, P.; Martins, L.M.D.R.S.; Guedes da Silva, M.F.C.; Fernandes, A.R.; Luis, D.; Silva, A.; Santos, S.; Borralho, P.M.; Rodrigues, C.M.P.; *et al*. Cobalt and Zinc Compounds Bearing 1,10-Phenanthroline-5,6-dione or 1,3,5-Triaza-7-phosphaadamantane Derivatives - Synthesis, Characterization, Cytotoxicity, and Cell Selectivity Studies. *Eur. J. Inorg. Chem.* **2013**, 3651–3658.

(c) Silva, T.F.S.; Martins, L.M.D.R.S.; Guedes da Silva, M.F.C.; Fernandes, A.R.; Silva, A.; Borralho, P.M.; Santos, S.; C.M.P.; Pombeiro, A.J.L. Cobalt complexes bearing scorpionate ligands: synthesis, characterization, cytotoxicity and DNA cleavage. *Dalton Trans.* **2012**, 12888-12897.

(d) Silva, T.F. S.; Martins, L.M.D.R.S.; Guedes da Silva, M.F.C.; Kuznetsov, M.L.; Fernandes, A.R.; Silva, A.; Pan, C-J; Lee, J-; Hwang, B-J; Pombeiro, A.J.L. Cobalt Complexes with Pyrazole Ligands as Catalyst Precursors for the Peroxidative Oxidation of Cyclohexane: X-ray Absorption Spectroscopy Studies and Biological Applications. *Chemistry, an Asian Journal* **2014**, 1132-1143.

(e) Sutradhar, M.; Fernandes, A.R.; Silva, J.; Mahmudov, K.T.; Guedes da Silva, M.F.C.; Pombeiro, AJ.L. Water soluble heterometallic potassium-dioxidovanadium(V) complexes as potential antiproliferative agents. *J. Inorg. Biochem.* **2016**, 17-25.

(f) Silva, J.; Rodrigues, A.S.; Videira, P.A.; Lasri, J.; Charmier, A.J.; Pombeiro, A.J.L.; Fernandes, A.R. Characterization of the antiproliferative potential and biological targets of a trans ketoimine platinum complex. *Inorganica Chimica Acta* **2014**, 156-167.

(g) Mendo, A.S.; Figueiredo, S.; Roma-Rodrigues, C.; Videira, P.A.; Ma, Z.; Diniz, M.; Larguinho, M.; Costa, P.M.; Lima, J.C.; Pombeiro, A.J.L.; *et al*. Characterization of antiproliferative potential and biological targets of a copper compound containing 4′-phenyl terpyridine. *J. Biol. Inorg. Chem.* **2015**, 935–948.

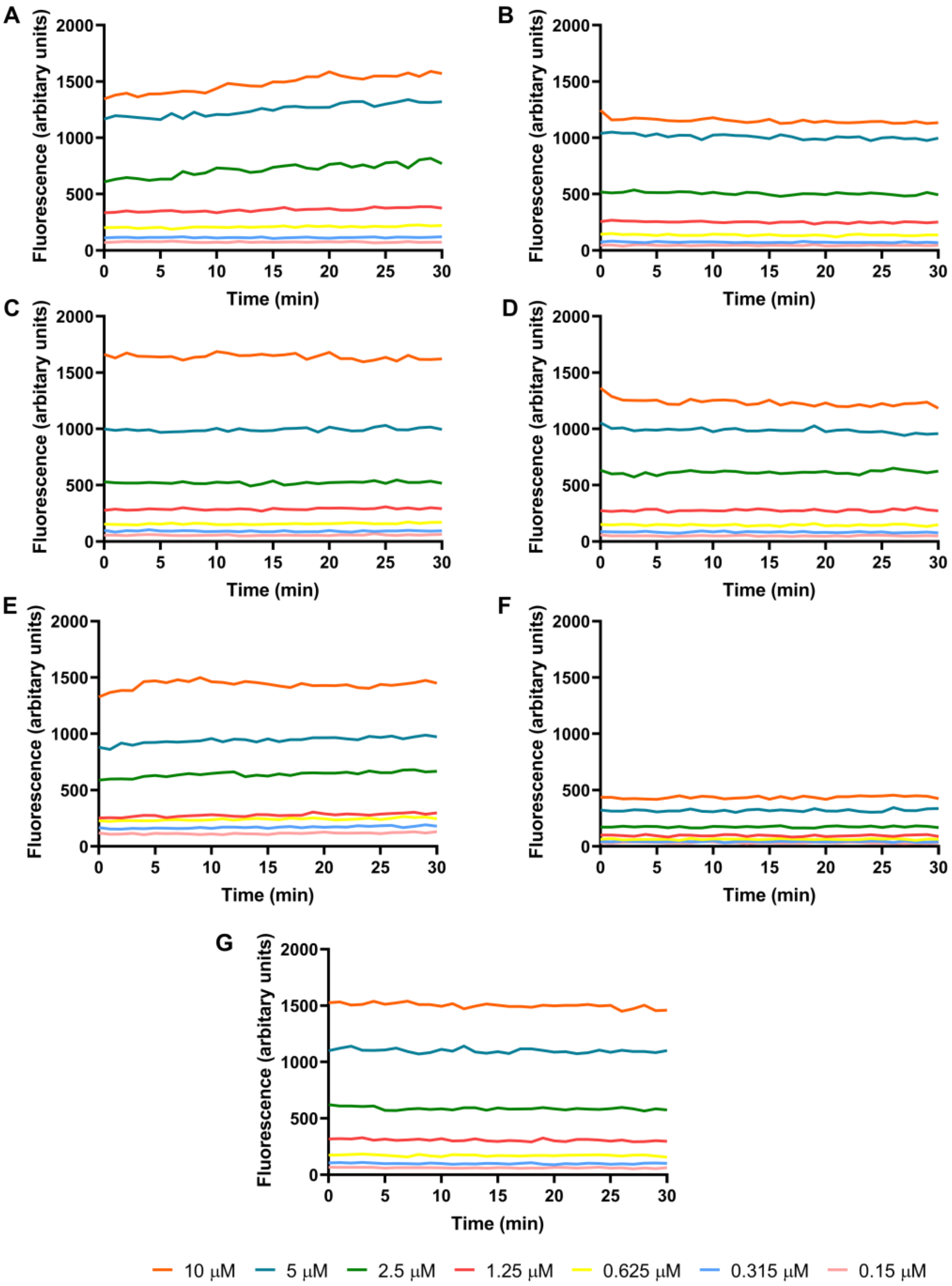

Figure S1. Determination of the equilibrium concentration of ethidium bromide (EtBr) in *S. aureus* (A), *L. monocytogenes* (B), *E. coli* (C), *S.* Typhimurium (D), *A. baumannii* (E), *K. pneumoniae* (F) and *P. aeruginosa* (G). Bacteria were incubated with a range of concentrations of EtBr during 30 minutes at 37°C. The fluorescence intensity of EtBr was recorded at excitation and emission wavelength of 515 and 600 nm. The results presented correspond to the average of 3 independent experiments.
